# Coastal sea level anomalies and associated trends from Jason satellite altimetry over 2002–2018

**DOI:** 10.1038/s41597-020-00694-w

**Published:** 2020-10-20

**Authors:** The Climate Change Initiative Coastal Sea Level Team, Jérôme Benveniste, Florence Birol, Francisco Calafat, Anny Cazenave, Habib Dieng, Yvan Gouzenes, Jean François Legeais, Fabien Léger, Fernando Niño, Marcello Passaro, Christian Schwatke, Andrew Shaw

**Affiliations:** 1 ESA/ESRIN, Frascati, Italy; 2 LEGOS, Toulouse, France; 3 NOC, Liverpool, UK; 4 ISSI, Bern, Switzerland; 5 OceanNext, Grenoble, France; 6 CLS, Ramonville St Agne, France; 7 TUM, Munich, Germany; 8 SKYMAT, Southampton, UK

**Keywords:** Physical oceanography, Ocean sciences

## Abstract

Climate-related sea level changes in the world coastal zones result from the superposition of the global mean rise due to ocean warming and land ice melt, regional changes caused by non-uniform ocean thermal expansion and salinity changes, and by the solid Earth response to current water mass redistribution and associated gravity change, plus small-scale coastal processes (e.g., shelf currents, wind & waves changes, fresh water input from rivers, etc.). So far, satellite altimetry has provided global gridded sea level time series up to 10–15 km to the coast only, preventing estimation of sea level changes very close to the coast. Here we present a 16-year-long (June 2002 to May 2018), high-resolution (20-Hz), along-track sea level dataset at monthly interval, together with associated sea level trends, at 429 coastal sites in six regions (Northeast Atlantic, Mediterranean Sea, Western Africa, North Indian Ocean, Southeast Asia and Australia). This new coastal sea level product is based on complete reprocessing of raw radar altimetry waveforms from the Jason-1, Jason-2 and Jason-3 missions.

## Abstract (summary)

**Table:** 

Measurement(s)	coastal sea level changes
Technology Type(s)	satellite imaging of a planet • computational modeling technique
Factor Type(s)	year of data collection
Sample Characteristic - Environment	coastal sea water • sea coast • ocean
Sample Characteristic - Location	Northeast Atlantic Ocean • Mediterranean Sea • West Africa • North Indian Ocean • Southeast Asia • Australia

Machine-accessible metadata file describing the reported data: 10.6084/m9.figshare.12999596

## Background & Summary

Since the early 1990s, sea level is routinely measured globally and regionally by high-precision altimeter satellites. The first high-precision altimetry measurements from space started with the launch in 1991 of ERS-1 by the European Space Agency (ESA), and in 1992 with the joint NASA (National Aeronautics and Space Administration) - CNES (Centre National d’Etudes Spatiales) satellite Topex/Poseidon (T/P). Since then, several altimetry missions have followed: Jason-1 (2001), Jason-2 (2008) and Jason-3 (2016), the successors of T/P with similar orbital characteristics. ESA also developed ERS-2 (1995), Envisat (2002), CryoSat (2010) and Sentinel-3A/3B (2016/2018). SARAL/AltiKa (2013), a joint Indian-French mission, operates in the Ka-band, allowing a smaller radar footprint on ground than other missions (T/P, the Jason series, ERS and Envisat being equipped with Ku-band radars). Cryosat and Sentinel-3A/3B use new technology, i.e. Synthetic Aperture Radar (SAR) altimetry. The T/P and Jason series have an orbital cycle of 10 days but a large spacing between satellite tracks (~300 km at equator). They cover the 66°S-66°N latitude domain. The orbital cycle of the ESA missions and SARAL/AltiKa is 35 days, but the spacing between tracks is smaller and the latitudinal coverage goes up to 82°, allowing a large portion of the Arctic Ocean to be covered.

**Fig. 1:**
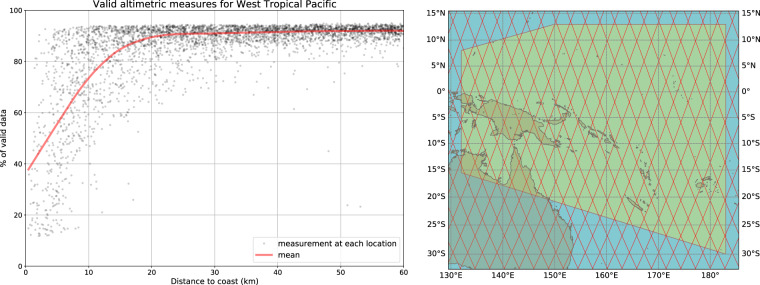
Percentage of valid range data for standard LRM altimetry missions, as a function of distance to the coast (left panel). Region (Western Tropical Pacific, green polygon) where the data come from (righ panel).

When combined, the current satellite altimetry record, 28-year long at the time of writing, shows that the global mean sea level is rising and even accelerating. Over the 1993–2019 time span, the mean rate and the acceleration amount to 3.3 +/− 0.3 mm/yr and ~0.1 mm/yr^2^ respectively^1–4^. Satellite altimetry also shows important regional variability in sea level trends, with rates up to 3 times the global mean in some regions^2,5,6^. While we now have precise sea level data sets from multi mission altimetry, at global and regional scales, this is not yet the case for coastal zones. This is because in classical altimetry sea level products, the amount of valid data strongly decrease within 10–15 km from the coast. An example is shown in Fig. 1 for data located in the Western Tropical Pacific. Classical criteria for declaring a data as invalid are: data on land, lack of one or more geophysical corrections, sea surface height (SSH) >1 m^7,8^.

**Fig. 2:**
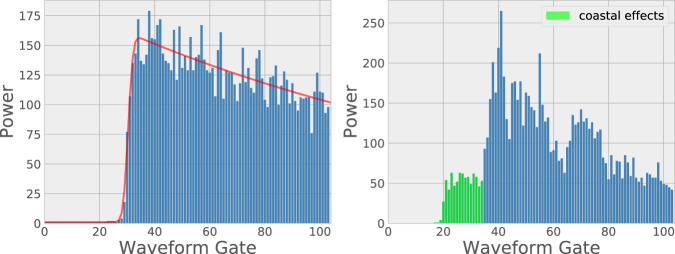
Typical altimetry waveforms along a Jason track in the Mediterranean Sea. Open ocean at location 43.02°N, 4.27°E (left panel). Coastal zone at location 43.34°N, 4.54°E (right panel).

This loss in valid sea level data close to the coast first results from land contamination within the footprint of Ku-band nadir altimeters (hereinafter called LRM for ‘Low Resolution Mode’). When the satellite approaches the coast, the radar signal reflected from the sea surface is modified in a complex way and differs from the standard Brown-type ocean waveform (the magnitude and shape of the radar altimetry return echo after reflection on the sea surface), preventing accurate estimation of the altimeter range (the distance between the satellite and the Earth surface measured by the onboard radar) used to estimate the sea surface height (also called sea level). This is illustrated in Fig. 2 showing a standard radar waveform over the open ocean and an example of waveform in coastal zones.

In addition, the geophysical corrections to be applied to the altimetry range measurements are usually optimized for the open ocean and not for the coastal zones. In the coastal zones, the wet tropospheric correction, based on on-board microwave radiometers is inaccurate at distances shorter than 20–50 km from land as land reflections enter the radiometer footprint. Tides and the high-frequency sea level response to wind and pressure changes need to be removed from altimetry data in order to avoid aliasing errors. However, in global models used to compute the corresponding geophysical corrections, significant errors still exist in coastal and shelf areas. The sea state bias (SSB) correction is usually estimated from models optimized for open-ocean altimetry measurements and becomes inaccurate within 10–15 km from the coast. More details can be found in^9,10^. Hence, the geophysical corrections applied to altimetry measurements remain a source of significant errors in the coastal zones. Note that the increased along-track resolution of SAR altimeters are able to provide more accurate ocean range measurements in the 0–10 km coastal zone, but even there, inadequate geophysical corrections remain a strong limitation as far as the accuracy of the sea level product is concerned.

Why is it important to precisely measure sea level in the coastal zone? Sea level rise is considered to be a major threat of current global warming to the low-lying coastal regions of the world. Coastal zones are indeed densely populated and concentrate important infrastructures such as harbors and industries. While the causes of the global and regional sea level changes are now well known and reasonably well quantified^3,4,6,11,12^, additional small scale processes of oceanographic and hydrological origin, specific to coastal areas, may superimpose on the global and regional components, eventually modifying the rate of sea level rise close to the coast compared to the open ocean^13,14^. Potential coastal process able to modify sea level trends at the coast include local atmospheric effects, baroclinic instabilities, coastal trapped waves, shelf currents, waves, fresh water input from rivers in estuaries. Hence, sea level change at the coast is not necessarily an extrapolation of the regional sea level trends that are routinely provided by standard ocean altimetry products^15^.

**Fig. 3:**
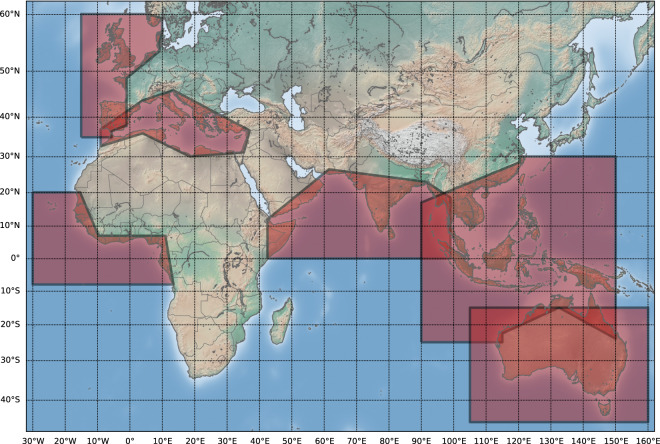
The 6 regions (in red) where new coastal sea level products are presented in this study.

About a decade ago, the European Space Agency (ESA) implemented the Climate Change Initiative programme (http://cci.esa.int/) dedicated to provide long, accurate and stable time series of a set of Essential Climate Variables (ECVs) observable from space, including sea level. The CCI sea level project (www.esa-sealevel-cci.org/) provided monthly gridded sea level maps over 1992–2015 based on the complete reprocessing of nine different altimetry missions, using new, optimized algorithms and geophysical corrections. In the context of additional activities, the CCI + Sea Level project, has proposed to extend the processing efforts to the coastal zones, and develop a new coastal sea level product in six selected regions: Northern Europe, Mediterranean Sea, Western Africa, North Indian Ocean, Southeast Asia and Australia (Fig. 3). This is a first step selection due to limited resources. Near future work will include the whole African coastlines. In case of project extension, the North and South America coastlines will also be studied.

The reprocessing method is described in Section 3. It consists of: (1) using an adapted retracking methodology to estimate the altimeter range from waveforms collected in the coastal zone, (2) using improved geophysical corrections, and (3) applying a strict data editing procedure adapted to coastal ocean conditions. The approach has been applied to the high frequency (20-Hz, corresponding to a ground resolution of ~300 m) along-track measurements from the Jason-1, Jason-2 and Jason-3 missions. For each satellite track, the sea level data of each mission have been combined into a single record over the study period (June 2002 to May 2018), and further expressed in terms of sea level anomalies located along a theoretical mean reference track. At all 20 Hz points along the track, the original sea level anomalies at 10-day interval have been further averaged on a monthly basis. Post-validation based on severe criteria (see section 4.2) has led to retain 429 coastal sites.

The corresponding regional product available is presented in section 4. It consists of: (1) monthly sea level anomalies time series at each 20-Hz point along the track, from June 2002 to May 2018, from 20 km offshore to the coast, and (2) sea level trends estimated over the study period at each 20 Hz point along the 20 km-long track portion.

## Methods

### Stage 1: The ALES retracking

The fitting of the altimetric waveform (retracking) in the open ocean is performed considering the full averaged echo registered on board the satellite at the typical frequency of 20 Hz. In the coastal zone, at distances up to about 20 km from land, the echoes are often corrupted by the presence of interferences in the trailing edge of the return. These can derive from the land intruding in the reflection, but also from the presence of areas with inhomogeneities in the backscatter characteristics of the illuminated surface.

In the Adaptive Leading-Edge Subwaveform (ALES) retracker, the retracking of each waveform is performed in two passes. A first pass looks at the rising portion of the waveform and provides a rough estimate of the significant wave height (SWH) from the slope of that portion. This estimate is then entered into an algorithm that selects the sub-waveform (i.e., sets the width of the fitting window over which a fitting is performed in the second pass). The dependence on the SWH is necessary to maintain the same level of precision achievable in the open ocean using a full-waveform retracker, given the direct relationship between sea state and noise of the retrieval. Full details of the retracking procedure are given in^16^.

Sea surface height and sea state are also related by the SSB correction. This correction is needed to correctly estimate the distance between the satellite and the mean surface of the illuminated area (Range). The SSB is determined by empirical models that relate SSH errors (for example differences at the intersection between two tracks) with the measured sea state. Since the sea state is measured using the same retracking method adopted to estimate the Range, any correlated error in the estimation of these parameters falls into the modelling of the SSB. The SSB corrections in the standard products are computed at low frequency (1-Hz, equivalent to an along-track distance of about 7 km), but correlated errors in the retracking affect the measurements at 20-Hz^17^.

In this study, an SSB correction is computed for every 20-Hz measurement applying the model developed by Tran *et al*.^18^ to the sea state retrievals of the ALES retracker. This approach has been demonstrated to improve the precision of the measurement by decreasing the variance of the SSH differences at crossover points by 10 to 20% depending on the region^19^. This improvement is due to the fact that the computation of a high-frequency SSB reduces the correlated errors between the retracked parameters^20^. The general aim of future research on SSB shall be to work on a retracked dataset that is free from the retracker-related noise, in order to correct for the physical effects of the interaction between the radar signal and the waves. Meanwhile, this approach offers a first step to improve the correction currently used in the standard products of the missions considered in this study.

The advantage of the ALES retracker coupled with the recomputed SSB correction in improving the quality and quantity of the altimetric records has been studied in recent years in several regional studies. The retracked SSH time series show a strong abatement in the number of outliers in the Baltic Sea/North Sea transition zone compared to the global along-track Sea Level CCI dataset^21^. Chereskin *et al*.^22^ have shown that the spatial spectrum of the SSH for the Jason missions retracked by ALES is comparable to the one obtained from Sentinel-3 measurements, despite the latter having an intrinsically better signal-to-noise ratio. The application of the retracker allows for a better estimation of the along-track mean sea surface profiles, as proved in the Gibraltar Strait^23^, and an improvement in the computation of the tidal constants from altimetry data in the coastal zone^24^.

### Stage 2. XTRACK-ALES Merging; Geophysical corrections; Editing

In this section we explain and describe the processing and post-processing system that has been implemented to derive the CCI + sea level products. The objective is to produce, from the combination of measurements provided by different altimetry missions, homogeneous long-term sea level time series and associated trends, as close as possible to the coastline (i.e. a few kilometers).

The X-TRACK/ALES processing and post-processing system is largely built on the X-TRACK software^7^, developed at the LEGOS laboratory (http://www.legos.obs-mip.fr/) in order to optimize the completeness and the accuracy of the SSH derived from satellite altimetry in coastal ocean areas. It is tailored for extending the use of altimetry data to coastal ocean applications and already provides freely available 1-Hz (i.e. with a resolution of 6–7 km in the along-track direction) sea level time series covering all the coastal oceans, distributed by the AVISO + operational centre. It is based on the standard ocean MLE4 (Maximum Likelihood Estimator 4) altimeter waveform retracker. The X-TRACK system is described in detail in^7,25,26^ (see also https://www.aviso.altimetry.fr/index.php?id=3047).

**Fig. 4:**
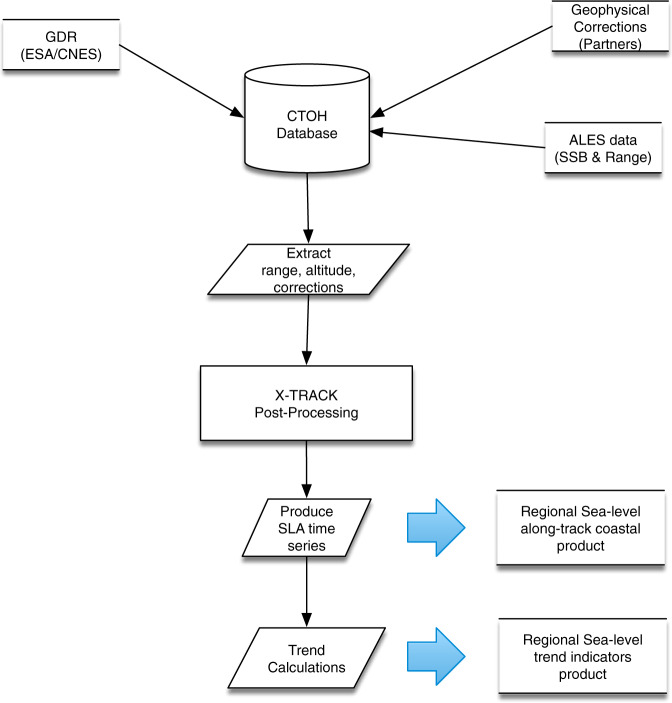
Sketch of the procedures applied to derive the coastal CCI + sea level products from altimetry measurements and geophysical corrections.

In the context of the ESA SL_CCI + project, X-TRACK has been extended to the processing of high-rate altimetry measurements (20-Hz in the case of Jason missions) instead of the 1-Hz data and then adapted to the ingestion of data provided by the ALES retracker. The resulting new processing system, called X-TRACK/ALES is illustrated by Fig. 4.

Like X-TRACK, the X-TRACK/ALES processing system works on a regional basis (the regional domain can be easily defined before the processing in a parameter file). It first uploads all the parameters needed to compute the product (orbit solution, altimeter ranges, instrumental, environmental and geophysical corrections), which were previously ingested and indexed in the altimetry database of the French National Observations Service for altimetry called CTOH (http://ctoh.legos.obs-mip.fr/). These parameters come from the Geophysical Data Records (GDRs) data sets distributed by the space agencies for the different altimetry missions, ALES products from TUM (Technical University Munich) and additional geophysical corrections provided by the RADS (Radar Altimetry Database System) altimeter database (http://rads.tudelft.nl/rads/rads.shtml) and the University of Porto for the GPD (GNSS-derived Path Delay) plus wet tropospheric correction^27^. In terms of altimetry corrections, the best altimeter standards defined in the ESA SL_cci project were selected in order to calculate sea level anomalies for climate studies (Table 1).

**Table 1:** List of altimetry parameters and geophysical corrections used in the computation of the SL_cci + coastal sea level product.

Parameter	Source	Jason-1/Jason-2/Jason-3
Altitude	GDR	Altitude of satellite
Range	ALES	20 Hz Ku band ALES corrected altimeter range^16^
sigma0	ALES	20 Hz Ku band ALES altimeter sigma0^16^
Ionosphere	GDR	From dual-frequency altimeter range measurement
Dry troposphere	GDR	From ECMWF (European Centre for Medium-Range Weather Forecasts) model
Wet troposphere	University of Porto	GPD + correction^27^
Sea state bias	ALES	Sea state bias correction in Ku band, ALES retracking^19^
Solid tides	RADS	From tide potential model^38,39^
Pole tides	GDR	From^40^
Loading effect	RADS	From FES 2014^41^
Atmospheric correction	RADS	From MOG2D-G^42^ + inverse barometer
Ocean tide	RADS	From FES 2014^41^

As already mentioned above, the characteristics of ocean altimetry measurements and corrections change significantly when approaching the coast, resulting in an important degradation of the corresponding sea level products (see^28^ for a complete review of the technical and processing issues related to coastal altimetry). To cope with this, the X-TRACK/ALES system first selects valid ocean data through a precise land mask (based on the GSHHS -Global Self-consistent, Hierarchical, High-resolution Geography- shoreline database distributed by NOAA, http://www.ngdc.noaa.gov/mgg/shorelines/data/gshhs/version2.1.1/) and a dedicated editing strategy. The latter includes two steps. The first step is to impose editing criteria, both on the altimeter measurements and corrections, designed to be more restrictive than the standard criteria used for the open ocean (https://www.aviso.altimetry.fr/fileadmin/documents/data/tools/hdbk_L2P_all_missions_except_S3.pdf). For each cycle, the spatial behavior of each correction is analyzed along the track, taking into account its individual characteristics. Abrupt changes are assumed to be associated with erroneous data and are removed^7^. In a second step, all corrections are recomputed at the 20-Hz frequency through interpolation/extrapolation methods, based on valid data for each correction. This strategy is very efficient in recovering a significant amount of valid altimeter measurements that were flagged in the standard product because of a deficient correction^25,29^.The corrected SSHs are computed at 20 Hz along-track point using Eq. 1, and are further projected onto fixed points along a nominal ground track and converted into Sea Level Anomalies (SLA) by subtracting a precise Mean Sea Surface (MSS) height using Eq. 2. The MSS is computed at the fixed nominal points, by inversion of all the available SSH measurements along the repeated ground tracks of the considered altimetry mission. This procedure allows better solving the coastal MSS gradients than the use of a standard gridded MSS product, thus reduces the errors in coastal SLA data^25^.

1 $${\rm{Corrected}}\,{\rm{SSH}}={\rm{Orbit}} \mbox{-} {\rm{Range}} \mbox{-} {\rm{Sum}}\,{\rm{corrections}}$$

2 $${\rm{SLA}}={\rm{Corrected}}\;{\rm{SSH}} \mbox{-} {\rm{MSS}}$$

The corrections account for atmospheric effects (wet and dry troposphere, ionosphere, inverse barometer), geophysical phenomena (ocean tides, high frequency atmospheric effects on the ocean) and the sea-surface state (electromagnetic sea-surface bias).

At this stage of the processing, we obtain a regional dataset of SLA time series with a temporal resolution of 10 days and a spatial resolution of ~300 m along the tracks of each Jason altimetry mission. The computation of a single long-term multi-mission product requires the application of inter-mission biases in order to remove instrument and corrections biases, thus to obtain stable merged sea level time-series. Inter-mission biases are computed during the “calibration phases” between two consecutive missions (about 3 month-long between Jason-1 and Jason-2 in 2008, and between Jason-2 and Jason-3 in 2016), when both satellites measure the same sea level on the same ground track, with about 1 minute time lag. The biases are computed for each of the six studied regions, excluding altimetry points located at less than 10 km from the coast, as well as points where more than 20% of data are missing in the time series. In order to remove the high-frequency variations in the resulting 20-Hz along-track bias values, known to be associated with measurement noise, the data are low-pass filtered (with a 40-km cutoff frequency) and averaged over 1° × 1° boxes. The corresponding smoothed 1° × 1° bias values are then interpolated at the original 20-Hz along-track altimetry points and applied to the SLAs. The Jason-1/2 inter-mission bias is applied to Jason-2 SLAs first, and then the Jason-2/3 inter-mission bias to Jason-3 SLAs.

It is worth mentioning that no orbit error reduction has been applied to the coastal sea level product. This is because such a correction based on differences between ascending and descending satellites tracks due to orbit errors needs to be computed globally. However, the orbit error is supposed to be small at the coast because of its large-scale behavior.

Finally, the long-term multi-mission SLA time series are monthly averaged, and a linear trend v and associated error are derived at each 20-Hz along-track point (see section 4.2 for details on the trend calculation).

A first version of the processing system described above has been successfully evaluated and validated in^30,31^. It significantly increases the number of valid SLAs in the coastal zone. As an example, in the Mediterranean Sea, 80% of valid sea level data are still available at distances of <2 km from coast, compared to distances of several km in the standard X-TRACK product^30^.

## Data Records

**Fig. 5:**
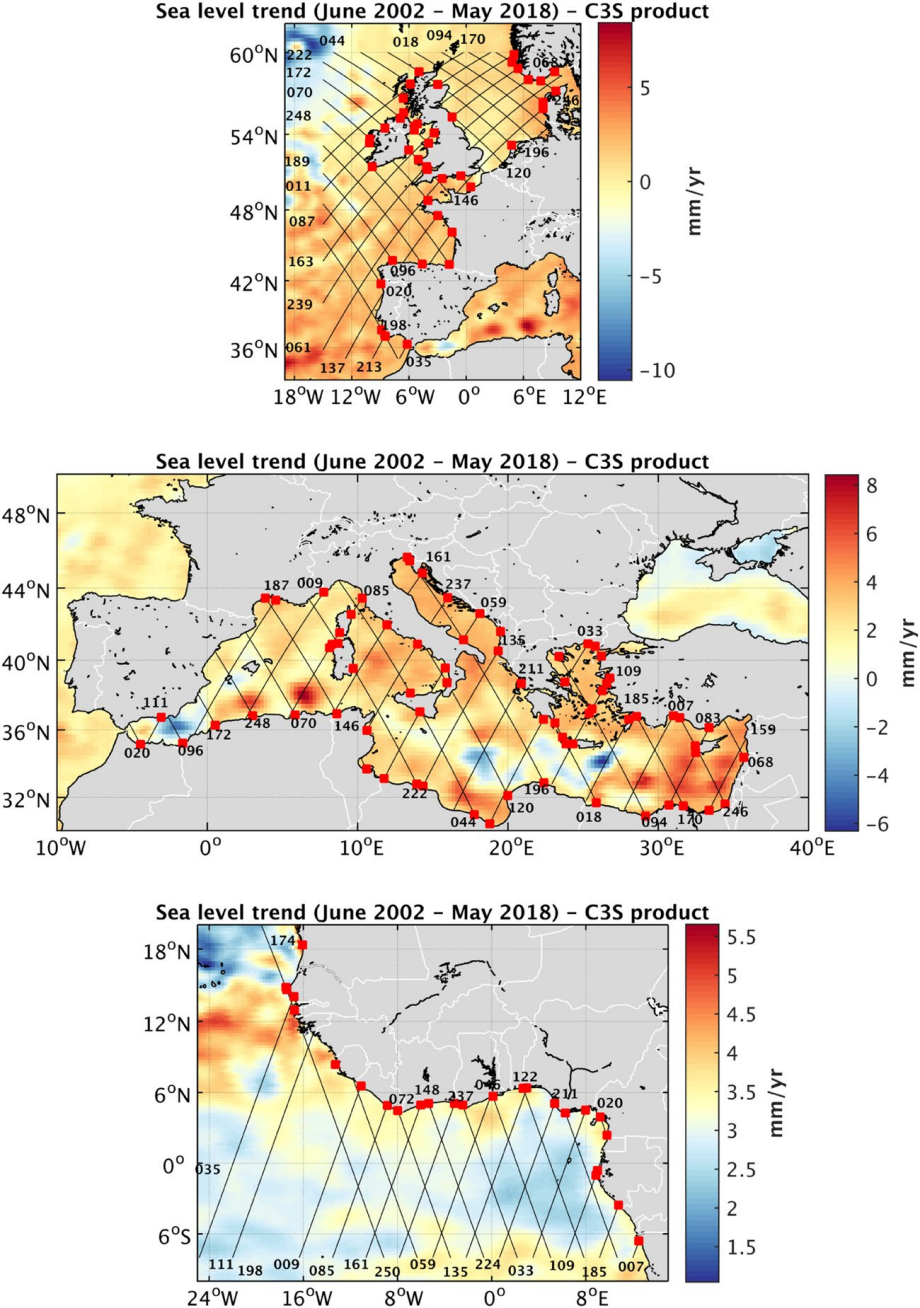
Jason tracks coverage (black lines, identified by numbers) superimposed on the sea level trend patterns over June 2002 - May 2018 (from the C3S data base, https://climate.copernicus.eu/). From top to bottom: Northeast Atlantic, Mediterranean Sea, Western Africa. Red squares represent the selected sites (see text).

**Fig. 6:**
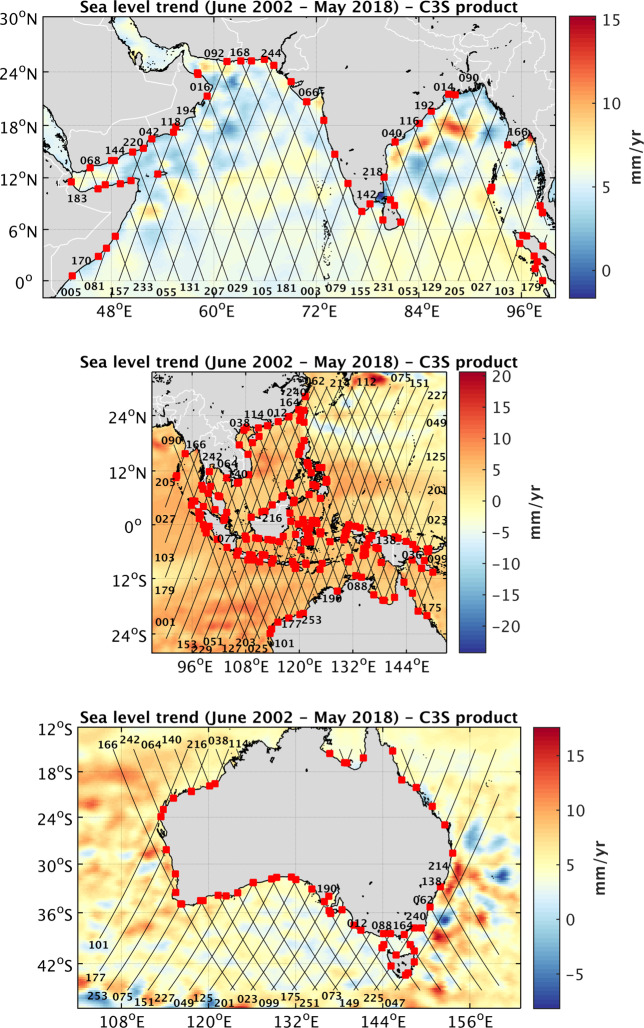
Jason tracks coverage (black lines, identified by numbers) superimposed on the sea level trend patterns over June 2002 - May 2018 (from the C3S data base, https://climate.copernicus.eu/). From top to bottom: North Indian Ocean, Southeast Asia, Australia. Red squares represent the selected sites (see text).

Using the regional data processing system described above, the measurements of the three Jason-1, Jason-2 and Jason-3 altimetry missions are combined in single homogeneous 10-day SLA time series over the period June 2002 – May 2018. These 10-day time series at each 20-Hz point, from 20 km offshore to the coast, represents the basic SLA data set called ‘**Coastal Sea Level Product 1**’^32,33^. It is available to users and distributed as NetCDF files. This data set contains 628 portions of 20 km-long tracks, crossing land at different locations across all regions. The ‘Coastal Sea level Product 1’ is not subject to any other further editing than the one done during the X-TRACK/ALES processing. The Jason track coverage for Northeast Atlantic, Mediterranean Sea and West Africa on the one hand, and for North Indian Ocean, Southeast Asia and Australia on the other hand, is presented in Fig. 5 and Fig. 6 respectively.

We further constructed another edited product called ‘**Coastal Sea Level Product 2**’ (delivered with this paper), that not only includes SLA time series (expressed as monthly averages), at each 20-Hz point along a track portion of 20 km from the coast, but also sea level trends and associated standard errors. This ‘Coastal Sea Level Product 2’ contains a much smaller number of track portions because based on a severe data selection, largely based on trend estimates (see below). The product includes monthly SLAs, simply computed by averaging available 10-day data (the monthly SLAs are based on a maximum of 4 values but sometimes only 2 or 1 value are available in the monthly interval). At each along-track 20-Hz point from 20 km offshore to the coast, annual and semi-annual signals were removed by fitting sinusoidal functions to the SLA time series. An editing was further applied by computing the mean of the deseasonalized and detrended SLA time series, and removing outliers located outside a 2-sigma threshold around the root mean squares of the time series (as in^31^). After re introducing the initial trend, a new trend and its associated 1-sigma formal error were estimated through a least-squares fit of a linear function to the edited SLA time series. Note that we do not correct the SLA time series for the regional contribution of Glacial Isostatic Adjustment. Such a correction is small (<1 mm/yr) and can be further removed from the estimated trends by the users.

The criteria considered for the selection of the monthly SLAs plus trends of the **Coastal Sea Level Product 2** are: (1) Number of valid data of the SLA time series at each 20-Hz points (missing data <50%); (2) Distribution of the valid data as uniform as possible across the three Jason missions. In a number of cases, Jason-1 data were much less numerous than the Jason-2 data. The corresponding SLA time series were then discarded; (3) Trend values in the range –15 mm/yr to +15 mm/yr (this threshold is based on occasional spurious discontinuities observed in sea level trends from one point to another, on the order of 10–15 mm/yr); (4) Standard errors on trends <2 mm/yr; (4) Continuity of trend values between successive 20 Hz points. Too abrupt changes in trends over very short distances were considered as spurious and the corresponding point was removed. This mostly occurred close to the coast but sometimes, at a larger distance from the coast.

With this selection approach, we discarded a large number of along-track 20-Hz points considered as not accurate enough to compute reliable trends. This led us to retain only 429 track portions from the initial set of 628 original track portions. We further identified them by a coastal site where the satellite track crosses land. The selected sites are named by the region and the track number to which they belong and by the site number on the track, going from north to south.

**Fig. 7:**
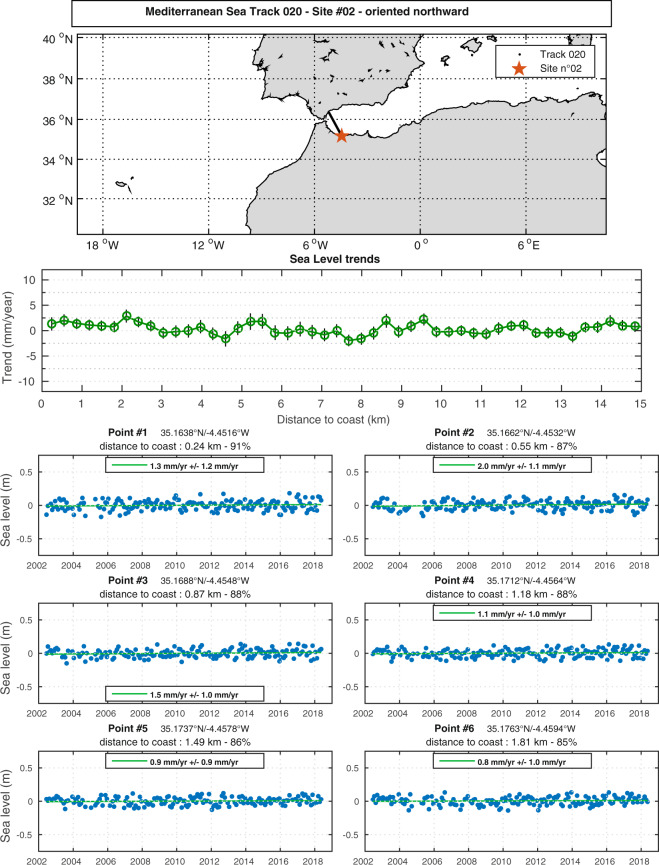
Position of the site n°2 on track 20 in Mediterranean Sea (red star, top panel), along-track sea level trends and trend errors (vertical bars) against distance to the coast (middle) and sea level anomalies time series of the 6 first points closest to the coast (bottom).

Figure 7 shows such an example, here site n°2 on track 20 in the Mediterranean Sea. From top to bottom, it shows a map of the site position on the track, the sea level trends at each 20-Hz point, expressed as a function of distance to the coast, starting from 15 km offshore, and superimposed trend error (shaded area), and finally SLA time series at the first six valid points from the coast.

The ‘Coastal Sea Level Product 2’ is available from the SEANOE repository website^34^.

The parameters provided to users at each 20-Hz point on a selected track portion are gathered in Table 2.

**Table 2:** List of altimetry parameters included in the ‘Coastal Sea Level Product 2’.

Variables	Description
lat	Latitude of each 20 Hz point
lon	Longitude of each 20 Hz point
distance_to_coast	Distance (m) to a reference point at the coast of each 20 Hz point
time	Time of measurements (days since 1950,0)
sla	Monthly Sea Level Anomalies (m) from X-TRACK/ALES 20 Hz
local_msl_trend	Sea level trend (mm/yr) computed from the monthly SLA
local_msl_trend_error	Sea level trend error (mm/yr)

## Technical Validation

### Statistics on coastal sea level trends

In this section, we present statistics on coastal sea level trends and associated trend errors, as well on distances to the coast of the first valid point. These results are shown in the form of histograms. For coastal trends and associated errors, separate histograms are provided for ascending and descending satellite tracks, as well as for ‘sea to land’ and ‘land to sea’ flying directions. The reason for looking at potential differences when the satellite flies from ‘sea to land’ or ‘land to sea’ is the following: The on-board tracking algorithm in the radar instrument suffers from some delay in adapting to the oncoming surface, therefore the radar is more efficient when it flies from a smooth surface to a harsh relief (sea to land) rather than the reverse. When it is tracking over land it needs up to one second after the land-sea transition to stabilize on the surface of the ocean.

**Fig. 8:**
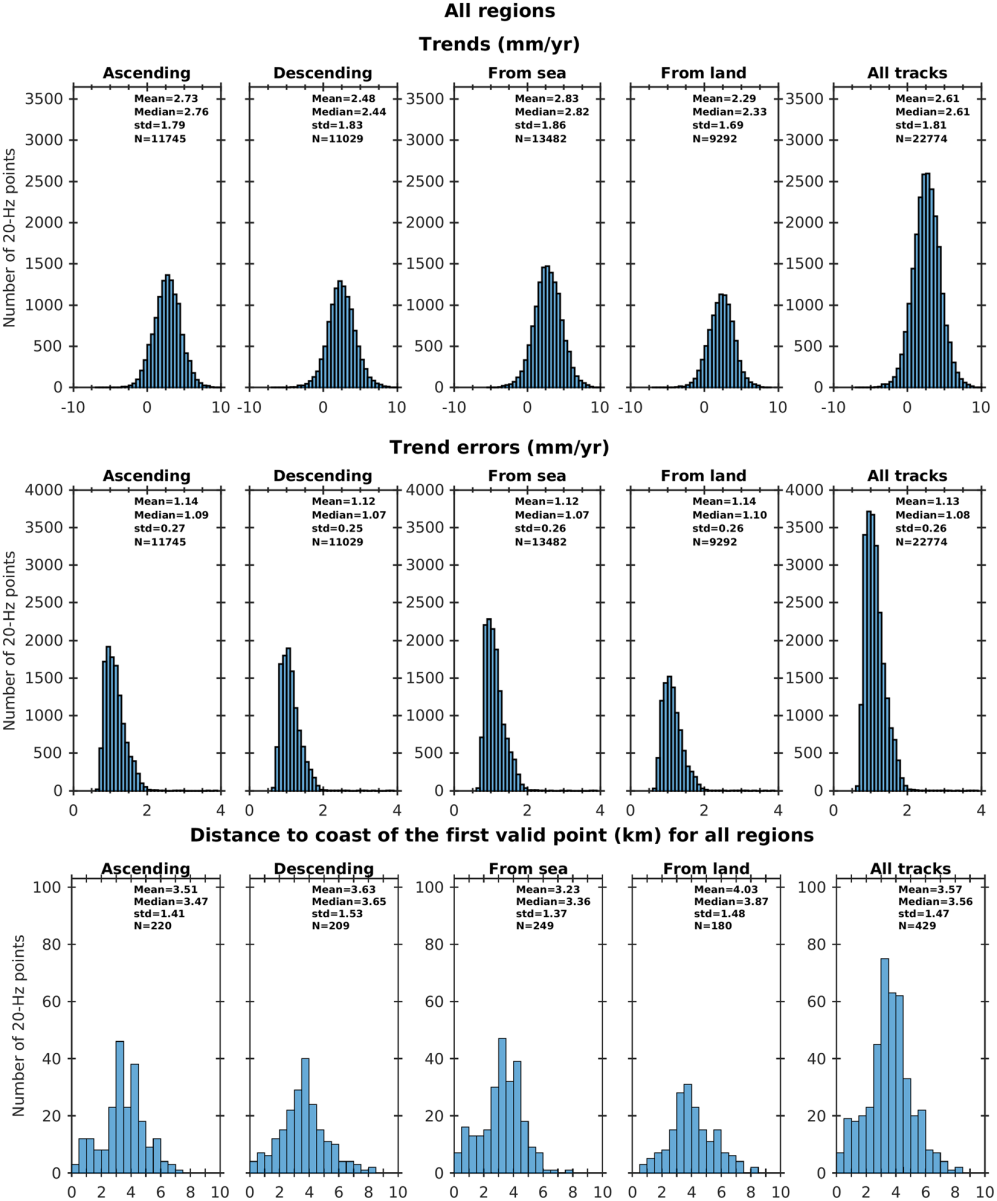
Histograms of trends (mm/yr) for ascending, descending, sea to land, land to sea and all tracks (upper panel) for all regions. Histograms of associated trend errors (mm/yr) (middle panel). Histograms of distance to coast of the first valid point (km) for ascending, descending, sea to land, land to sea and all tracks (all regions) (lower panel).

Similarly, histograms of closest distance to the coast of the first valid point are presented for ascending and descending satellite tracks, as well as for ‘sea to land’ and ‘land to sea’ flying directions. In all cases, this is done for all six regions together and for each region individually. Results are presented in Fig. 8 for all six coastal zones.

The histograms shown in Fig. 8 display no differences between ascending and descending tracks in terms of trends distribution. Although it has to be noted that the sites where the track crosses land are different for ascending and descending tracks, the distributions looks quite similar in both cases and mean trend values are on the same order of magnitude within 0.1 mm/yr. The mean value for all tracks amount to 2.6 mm/yr. Note that this trend value is not GIA-corrected. Several GIA models exist and users can apply the GIA correction of their choice using available gridded GIA data sets. Because of differences between models, we prefer not to apply this correction.

The mean and median trend errors are also similar for ascending and descending tracks, and on the order of 1.1 mm/yr. Note that there is no trend error larger than 2 mm/yr, a consequence of one of our selection criteria (see section 4.2).

In Fig. 8 are also shown the distributions of the closest distance to the coast (first valid point) for the same configurations as for the trends. Again little difference is observed between ascending and descending tracks, with a mean distance of 3.5 km for all sites. On the other hand, we note better performance for the sea to land configuration (mean value of 3.2 km, more sites with closest distance to coast <2 km) than the land to sea configuration.

Similar histograms for individual regions (not shown) display some differences from one region to another. In the Mediterranean Sea, 70 sites are selected. Their mean coastal trend is 1.9 +/−1.0 mm/yr. No difference is noted between ascending and descending tracks. There is a smaller amount of land to sea configurations in the selected sites, with slightly smaller coastal trends (1.7 +/− 1. mm/yr) than in the sea to land case (coastal trends of 2. +/− 1. mm/yr). The distances of the first valid point to the coast are spread from <1 km to >5 km. The mean distance is in the 3–4 km range but a larger number of cases fall within less than 2 km from the coast. In the northeast Atlantic region, 44 sites are selected. The mean trend is 2. +/− 1.2 mm/yr. Only slight difference is observed between ascending (1.85 +/− 1.2 mm/yr) and descending tracks (2.05 +/− 1.2 mm/yr). Five time more measurement points are seen for the sea to land configuration than the land to sea one. The mean distance of the closest valid point is 3.5 km, with only few cases <2 km and most of the distribution lying between 2 km and 4 km.

26 sites only are selected along the Western Africa region. The mean rate of coastal sea level rise is 2.15 +/− 0.9 mm/yr. Along ascending tracks, the mean trend is 2. +/− 0.9 mm/yr while it is only 1.35 +/− 0.9 mm/yr along descending tracks. But the latter concerns a much smaller number of measurement points. We also observe more sea to land cases than land to sea, due to the particular configuration of the African coast and Jason tracks. On average, the closest distance to coast distribution is in the 2–4 km range, with most of the cases included between 3 and 4 km to the coast. In the northern Indian Ocean region, 57 sites have been selected. The average distance to coast of the first valid point lies in the 3–4 km range but we observe a large spread from <1 km to >5–6 km from the coast. The mean trend is 3.5 +/− 1. mm/yr, with no noticeable difference between ascending and descending tracks nor between sea to land and land to sea directions. The Southeast Asia region displays the largest number of selected sites (177), with a mean trend of 2.7 +/− 1.2 mm/yr. As for the north Indian Ocean region, no significant difference is seen between ascending and descending tracks nor between sea to land and land to sea directions. The mean distance of the closest valid point is 3.8 km, with a maximum of the distribution between 3 km and 4 km. Finally, 55 sites are selected around Australia. The mean trend is 3.2 +/− 1.2 mm/yr. In this region, several sites display sea level trends of 5 mm/yr or larger. We note more valid points for ascending than descending tracks. The mean value of the closest distance to coast is 3.3 km, with a more or less uniform distribution between the coast and 6 km offshore.

**Fig. 9:**
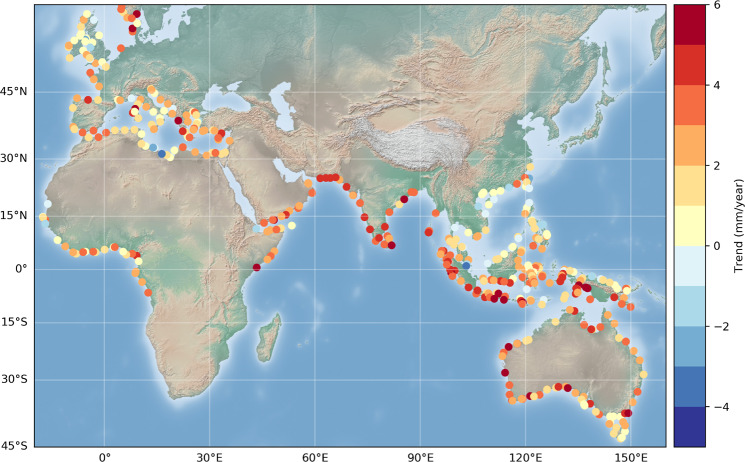
Coastal sea level trends (mm/yr) at the first valid point from the coast at the 429 selected sites.

A map of coastal trends (averaged over 2 km along-track from the first valid point) is shown in Fig. 9. The figure indicates that at a significant number of sites, over the study period, the coastal sea level rise is in general positive (with a few exceptions), with values as high as 4–5 mm/yr in some regions. This is particularly the case in the northern and eastern parts of the Indian Ocean (around Indonesia for the latter).

**Fig. 10:**
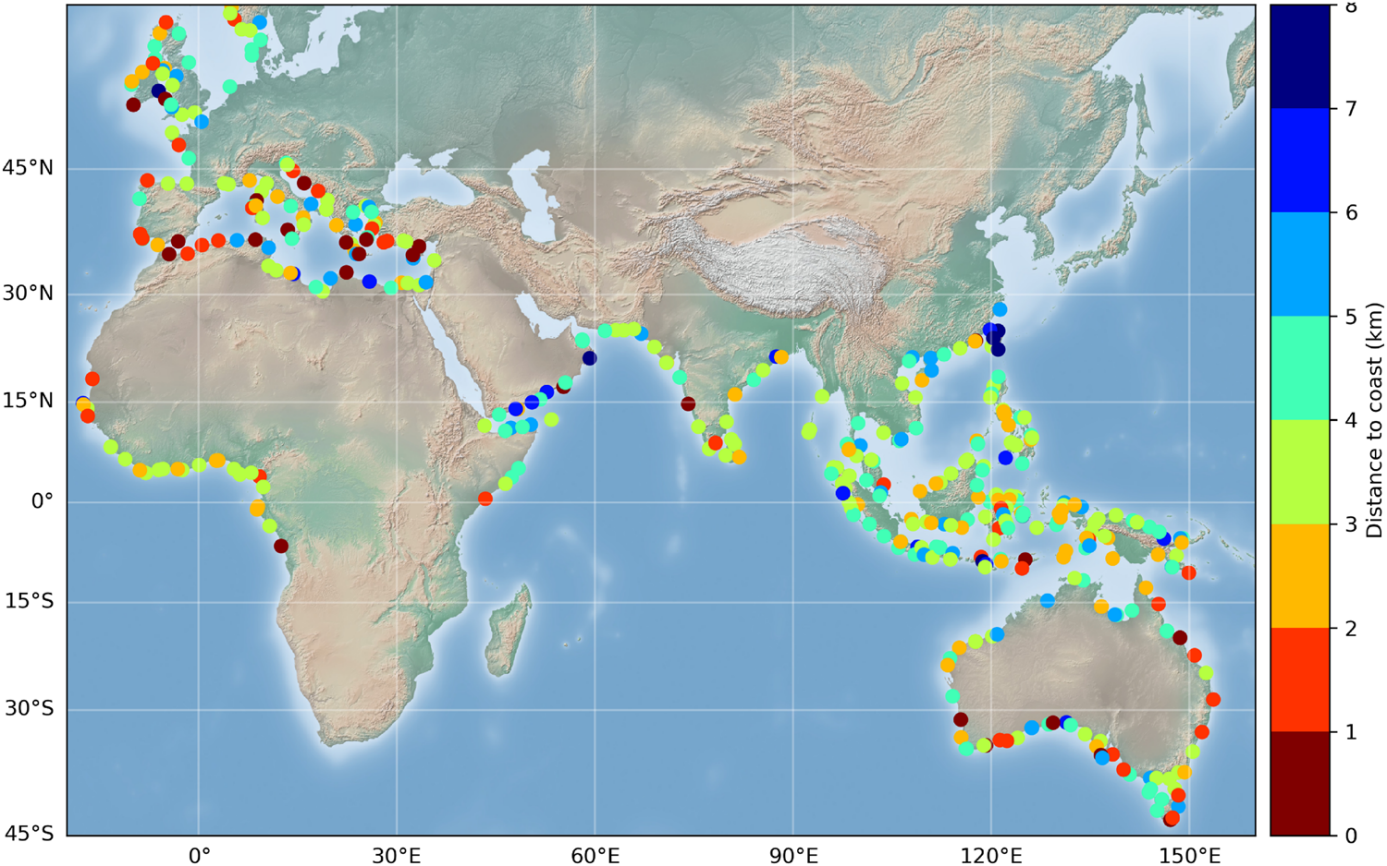
Map of the closest distance (km) to the coast of the first valid point from the coast at the 429 selected sites.

In Fig. 10 is shown a map of the distance to coast of the first valid point.

Figure 10 shows that in most regions, the distance to the coast of the first valid point in the 3–4 km range, but as discussed above, the closest distance to the coast can be <2 km, particularly in the Mediterranean Sea and around Australia.

**Fig. 11:**
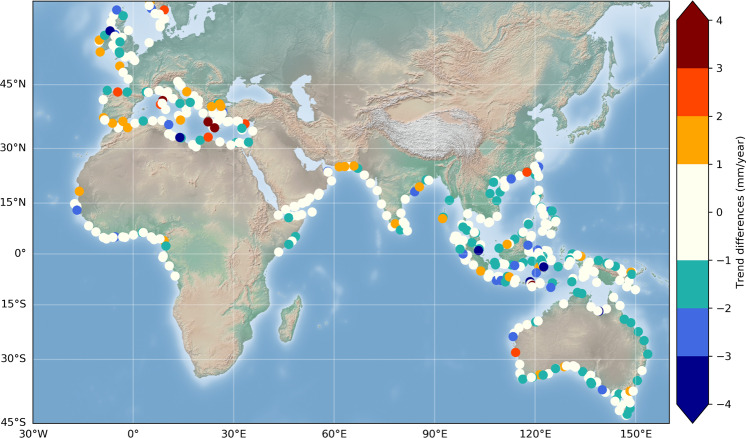
Differences in sea level trends between an along-track band of 2 km from the closest valid point to the coast and the 14–16 km average, offshore. White points correspond to no significant differences (within +/−1 mm/yr) between open ocean and coast. Orange-red/blue dots correspond to coastal trend increase/decrease at the coast.

In order to investigate whether the coastal trends shown in Fig. 8 differ from open ocean trends, the differences in sea level trends between an along-track portion of 2 km from the closest valid point to the coast and the 14–16 km average, offshore, have been computed. These are shown in Fig. 11.

The trend difference map presented in Fig. 11 shows an unexpected result: In most places, no significant difference (within +/−1 mm/yr) is noticed between the open ocean (here assumed ~15 km away from the coast) and the coastal zone (the first few km from the coast). However, this is not always true. At a few sites, we observe a larger trend close to the coast than offshore, but with the exception of 3 sites in the Mediterranean Sea and one site in Australia, the increase is modest, of 1–2 mm/yr only, and possibly not significant in view of the trend uncertainties. In a number of cases, we note a decrease in trend as the distance to the coast decreases (blue points on the map). But here again just a few cases may be significant. These particular sites are the object of an on-going study.

Although it had been expected that coastal processes may cause some discrepancy in coastal sea level trends compared to the open ocean^13,14^, the results presented here seem to contradict this hypothesis in about 80% of our 429 selected sites. An important consequence of this observation is that it would be possible to extrapolate up to the coast, regional sea level trends computed by classical altimetry missions. More investigations using additional satellites and longer records are definitely needed to confirm such results.

### Validation with tide gauges

In this section we present a comparison of the new altimetry product with tide gauge observations. The tide gauge data used here consists of monthly mean values of sea level spanning the same period as the altimetry data and are obtained from the Revised Local Reference data archive of the Permanent Service for Mean Sea Level (http://www.psmsl.org/)^35^. To be consistent with the altimetry data, the same atmospheric correction that is applied to the altimetry data (i.e., MOG2D-G + inverse barometer) is also applied to the tide gauge data. The comparison between the two types of measurements is conducted in terms of sea-level variability (detrended and deseasoned monthly time series of sea level) and trends over the period from June 2002 to May 2018. In designing the validation strategy, a number of points merit consideration.

First, it is important to recognize that while the tide gauge data represent true monthly mean values, the altimetry monthly data are based on at most four measurements per month (due to the 10-day orbital cycle of the Jason missions) and so such data will be subject to sampling uncertainty due to variability at sub-monthly timescales. This sampling uncertainty will manifest as differences with the tide gauge observations, both in the variability and the trend. Exploratory analysis of this issue (not shown here) indicates that the effect of sampling uncertainty is fairly small when using three or more values per month, however it is more noticeable when using only one value per month and can degrade the correlation between the two otherwise identical time series, on average, from 1 to 0.7 and cause trend differences as large as 1.5 mm/yr. It is important to keep these effects in mind when interpreting the results of the validation against tide gauge data.

A second point to note is that, in general, altimetry measurements are not taken at tide gauge locations but at some ocean point nearby, and this spatial separation will inevitably lead to differences between the two types of data. The importance of such differences will depend on the length scales of the sea-level signals around the tide gauges and can be significant. For this comparison, we first select the closest altimetry track to each tide gauge station and then, along this track, we select the altimetry time series showing the highest correlation with the tide gauge record. This altimetry time series is the one that we use in our comparison.

A third point to consider is that tide gauges measure sea level relative to the land on which they reside and so the measurements can be strongly affected by vertical land motion (VLM), particularly on long timescales. When comparing trends from altimetry and tide gauges it is important to account for this land contribution. Here, this is done by adjusting the tide gauge rates using Global Positioning System (GPS) vertical velocities. The GPS data consists of VLM rates from three different solutions (ULR, NGL, and JPL) and are obtained from SONEL (https://www.sonel.org). If there is a GPS station within 5 km of the tide gauge, then we use the rates from such station as our estimate of VLM (averaged over the various GPS solutions – ULR, NGL and JPL – available), otherwise we average the GPS rates over all GPS stations (and solutions) that are located within 50 km of the tide gauge. If there are no GPS stations within 50 km of the tide gauge, then we do not consider such tide gauge in the trend comparison.

The agreement between altimetry and the tide gauges in terms of trends is evaluated using fractional differences (FDs), which are defined as ${\rm{FD}}=\left|{\tau }_{d}\right|/(1.97* SE)$, where *τ*_*d*_ is the trend of the time series of sea level differences between altimetry and the tide gauge, *SE* is the associated standard error and 1.97 is the critical value of the Student’s t-distribution for the 95% confidence level. Hence, an FD value >1 means that, with 95% confidence, the two trends are statistically different. To be as rigorous as possible in the comparison of trends and obtain proper standard errors, we account for serial correlation in the estimation of the trends by using a regression model with first-order autoregressive errors. The model is analogous to that described by Chib^36^.

**Fig. 12:**
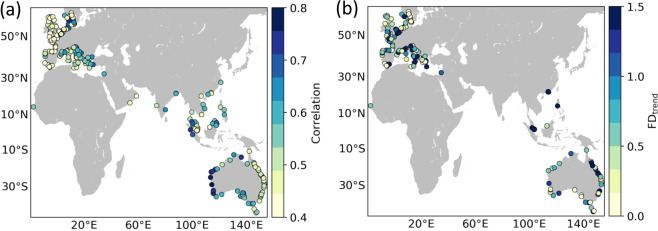
Correlations (**a**) and trend FDs (**b**) for the comparison between altimetry and tide gauge observations. The correlations are for detrended and deseasoned monthly timeseries. Only tide gauges with a GPS station within 50 km are shown in (**b**).

The average correlation between the altimetry and tide gauge time series across all tide gauge stations is 0.5, indicating an overall good match, but there are clear differences in agreement between regions (Fig. 12a). These differences are most obvious along the Australian coastlines, where correlations are significantly higher along the western coast (>0.7) than on the eastern coast (~0.5). This is indicative of sea-level signals with shorter length scales along eastern Australia resulting in larger differences due to spatial separation, and thus it should not be interpreted as reflective of a difference in altimetric performance between the two coastlines. Sampling uncertainty due to variability at sub-monthly timescales present in the altimetry time series is also likely to play a role in explaining the relatively low correlations in some regions.

To place the correlations shown in Fig. 12a into a broader context, we next compare those with the correlations obtained using the SSALTO/Duacs all-sat-merged gridded product distributed by CMEMS (https://marine.copernicus.eu) at the same tide gauge stations (we compare each tide gauge with the nearest gridded point). The average correlation for the CMEMS data is 0.74 across all tide gauges, which is indeed higher than the average correlation we find for point-level measurements in our product (0.51). However, the higher correlations found with the gridded altimetry product are to be expected because the gridding process alleviates the issue of sampling uncertainty (monthly means are based on more values) and reduces the influence of both small-scale variability and measurement errors (data are ‘averaged’ across space and over several altimetry missions).

To illustrate this last point, we perform a second comparison with tide gauge data using an approach that merges altimetry data from different tracks based on sea-level length scales. For this, we use the SL_cci + XTRACK-ALES v1.1 along-track coastal product (available at http://www.esa-sealevel-cci.org/products), on which the data presented in the paper is based (this product provides data beyond 20 km from the coast, which are needed for this approach). We first estimate coherence length scales of sea level variability at each tide gauge by first correlating the deseasoned and detrended sea-level from the tide gauge record with that from along-track altimetry, and then fitting a Matérn function^37^ to the vector of correlations as a function of distance to the tide gauge. Then, at each tide gauge, we merge the altimetry data from all tracks that fall within the estimated length scale into a monthly time series by averaging spatially along tracks and temporally across tracks. Using only altimetry data within a length scale from the tide gauge can reduce differences due to spatial separation. In addition, if more than one altimetry track falls within the estimated length scale, our approach allows us to compute monthly values based on many more than 4 values, alleviating the issue of sampling uncertainty. The average correlation between our merged altimetry time series and the tide gauge data is 0.78, which is slightly higher than the correlation for the CMEMS gridded product. Importantly, there are 19 tide gauge stations where our product gives significantly higher correlations (>0.2), suggesting that our product performs better at locations where sea level signals have relatively small length scales. Note that at locations where the CMEMS data performs better, the difference in correlation is smaller than 0.18 in all cases.

In regard to the trends (Fig. 12b), the median FD is 0.69 and FDs are <1 at 64% of the tide gauge stations, indicating that altimetry and tide gauge trends are in good agreement at the vast majority of stations. Again, there are regional differences such as the better agreement in western Australia compared to eastern Australia. This is again suggestive of shorter sea-level length scales along the eastern coast. There are 10 stations where FDs are >3, which reflects large trend differences. Such differences are likely due to local VLMs at the tide gauge stations that are not captured by the non-colocated GPS stations.

**Fig. 13:**
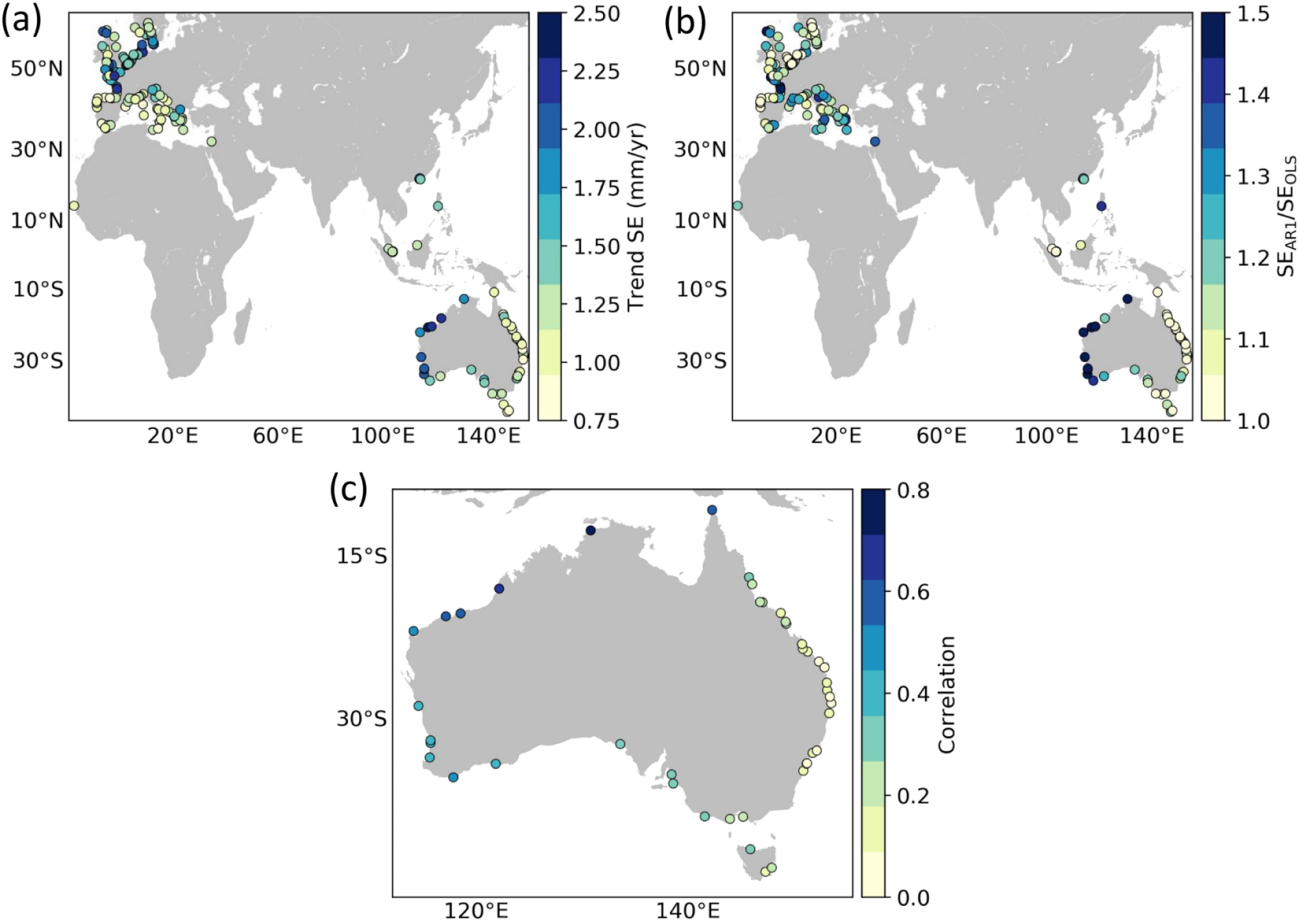
Standard errors associated with the tide gauge trends as estimated by a model that accounts for serial correlation (**a**), along with the ratio between standard errors with and without consideration of serial correlation (**b**). Standard errors that ignore serial correlation are computed using ordinary least squares (i.e., assuming the residuals from the regression model are normally distributed). (**c**) Correlation between the detrended and deseasoned tide gauge records and the Southern Oscillation Index.

To get a sense of the magnitude of the trend standard errors and the effect of serial correlation on such errors, we show the values of the standard errors at all of the tide gauge stations (Fig. 13a) along with the ratio between errors with and without serial correlation adjustment (Fig. 13b). The value of the standard errors ranges from ~0.75 mm/yr to ~2.5 mm/yr, with the largest values found in regions of relatively large sea-level variability such as the North Sea and the western coast of Australia (Fig. 13a). The degree of serial correlation also varies with region, with the largest effect found in western and northwestern Australia where the true standard errors (i.e., those that account for serial correlation) can be more than 50% larger than the errors given by ordinary least squares. Interestingly, serial correlation is almost negligible along the eastern coast of Australia. This contrast between the western and eastern coasts of Australia partly reflects the much larger influence of the El Niño - Southern Oscillation on sea level along the western coast of Australia, which results in large low-frequency fluctuations there (reddening the spectrum, hence increasing serial correlation) (Fig. 13c).

## Summary

In this paper, we have described a new coastal sea level product based on reprocessed satellite altimetry data from the Jason missions, available to users for a variety of applications, including studies of sea level change close to the coast and associated coastal impacts. This product is, to our knowledge, the first coastal sea level data set available at high along-track resolution (~300 m) over a time span longer than a decade. It includes validated sea level anomalies in the close vicinity of the coast (within 20 km from the coast) and associated coastal sea level trends in six regions. As shown in this study, it helps answering the question: is coastal sea level rising at the same rate as open ocean sea level? In the context of the on-going ESA CCI + project, we plan to extend in time and space this data product by updating the Jason-3 record and using additional satellites with smaller inter-track spacing (Envisat, SARAL/AltiKa, Sentinel-3A, Sentinel-3B). In this coming next phase, the data coverage will include coastlines of the whole African continent. On a longer time span, if resources permit, other regions will be studied, in particular North and South America. On the short-term, future activities will also be devoted to investigate which coastal processes cause departure of the rate of sea level change at the coast compared to the open ocean at the few sites where a trend increase/decrease has been reported (if *in situ* data and/or high-resolution hydrodynamical models are available). Finally, systematic comparisons with tide gauge trends will be performed at sites where the satellite tracks cross land in the vicinity of a tide gauge.

## Data Availability

The numerical code corresponding to the X-TRACK/ALES processing and post-processing system is not public. It is based on the merging of methodologies previously described in^7^ and^16^. Further code evolutions and associated data sources are indicated in the present manuscript.
